# Radiation boost for synchronous solitary inguinal lymph node metastasis during neoadjuvant chemoradiotherapy for locally advanced rectal cancer

**DOI:** 10.1007/s12672-021-00455-0

**Published:** 2021-12-04

**Authors:** Mo Chen, Shuai Liu, Meng Xu, Han-chen Yi, Yanping Liu, Fang He

**Affiliations:** 1 Radiotherapy Department of Thorax and Abdomen Carcinoma, Cancer Center, The First People’s Hospital of Foshan, Foshan, China; 2 Department of Radiation Oncology, The Sixth Affiliated Hospital of Sun Yat-Sen University, 26 YuanCun ErHeng Road, Guangzhou, 510655 Guangdong China; 3 Guangdong Provincial Key Laboratory of Colorectal and Pelvic Floor Diseases, Guangdong Institute of Gastroenterology, Guangzhou, China

**Keywords:** Solitary inguinal lymph node, Radiation boost, Neoadjuvant chemoradiotherapy, Rectal cancer, Synchronous

## Abstract

**Background:**

Some patients with locally advanced rectal cancer (LARC) present with inguinal lymph node metastases without evidence of other systemic disease, known as solitary inguinal lymph node metastasis (SILNM). These patients may represent a distinct subset who have a more favorable prognosis and should be treated with curative intent. The optimal treatment strategy for these patients has not been determined.

**Methods:**

We retrospectively reviewed 16 consecutive LARC patients diagnosed between January 2017 and December 2019, who had SILNM, were treated with an inguinal lymph nodes (ILN) radiation boost with curative intent during neoadjuvant chemoradiotherapy (nCRT) and underwent total mesorectal excision (TME). We used Kaplan–Meier survival curves to calculate survival rates, and recorded radiation-related toxicity.

**Results:**

None of these 16 patients developed pelvic or inguinal recurrences, and 3 of the patients developed distant metastases. The 3-year overall survival rate and locoregional relapse-free survival rate were both 100%. The 3-year disease-free rate and distant metastasis-free survival rate were both 81.3%. Of 5 patients who had ILN dissection for suspicious ILNs after neoadjuvant treatment, 2 had residual nodal tumor confirmed. Grade 3 toxicity was found in 5 patients, and no patients had lymphedema or other grade 4 or 5 toxicities.

**Conclusions:**

In LARC patients with synchronous SILNM, a radiation boost to the ILNs during nCRT achieved excellent local control with acceptable toxicity. Though the optimal treatment strategy remains unclear, nCRT with an ILN radiation boost prior to TME may be a reasonable therapeutic approach to consider for this subset of patients.

## Introduction

In patients with locally advanced rectal cancer (LARC), the presence of tumor in an inguinal lymph node (ILN) is now categorized as a metastasis (M +) in the 8th edition of the tumor-node-metastasis (TNM) classification, primarily because this finding is thought to represent systemic disease and is associated with a relatively poor prognosis [1]. ILN metastases (ILNM) from rectal cancer can be classified as synchronous, when they present concomitantly with the primary tumor and before surgery, or metachronous, when they occur after surgery [2].

ILNM from rectal cancer is unusual, particularly when it occurs without signs of distant spread to other organs or sites. ILNM arise from either proximal lymphatic obstruction secondary to bulky rectal tumor or retrograde spread related to extensive rectal tumor in the pelvis [3]. However, some patients present with ILNM without evidence of other systemic metastases, a phenomenon known as solitary inguinal lymph node metastasis (SILNM), and which may be the result of direct invasion into the inferior lymphatics in the distal rectum [4]. It appears that these patients can benefit from inguinal lymph node dissection (ILND), some even achieving long-term survival [4, 5]. Another treatment option for these patients involves giving a radiation boost to the ILNs as part of neoadjuvant chemoradiotherapy (nCRT), followed by surgery with a curative intent [6].

Patients with SILNM may represent a distinct subset of patients with metastatic rectal cancer who have a more favorable prognosis than those with other metastases [6]. Some have argued that these patients should be managed differently than most patients who have systemic disease; however, the optimal treatment strategy for these patients has not yet been identified.

In our hospital, we have recently been treating these patients with a radiation boost to a total dose of 58.0 Gy to metastasis ILNs, while they are receiving neoadjuvant chemoradiotherapy (nCRT), and before they undergo total mesorectal excision (TME). We have been performing TME alone if the ILN disappeared after nCRT (including the radiation boost); if the ILN have not disappeared, we have been performing TME plus ILND. For this retrospective study, we evaluated the outcomes and radiation-related complications in a group of patients with LARC and synchronous SILNM who underwent a radiation boost to the ILNs with curative intent as part of neoadjuvant chemoradiotherapy (nCRT), prior to undergoing TME with or without ILND.

## Materials and methods

### Patient selection

We retrospectively reviewed the cases of 16 consecutive patients who were diagnosed with biopsy proven LARC between January 2017 and December 2019, were noted to have biopsy-proven evidence of synchronous SILNM and underwent external beam radiation boost to the ILN with curative intent during nCRT, followed by total mesorectal excision (TME). For the purpose of our study, we defined synchronous SILNM as those which were identified concomitantly with the primary tumor and before surgery [2].

All patients included in the study met the following criteria: (a) clinical stage II (T3-4N0) or stage III (T1-4N1-2) rectal cancer, other than the presence of metastasis ILNs, and (b) no distant metastasis, both determined at the time of pretreatment evaluation by magnetic resonance imaging (MRI), computed tomography (CT), and/or endorectal ultrasonography (EUS). Clinically positive ILNs were defined as those that had a short-axis diameter of 8 mm or greater on MRI or CT, were positive on diffusion-weighted MRI, had irregular borders on MRI or CT, and/or presented as hot spots on PET [7]. All the patients with clinically suspicious ILNs underwent ultrasound guided biopsy and diagnosed as metastasis lymph node before treatment. Patients were excluded from the study if they had a histologic type of rectal cancer other than adenocarcinoma, had metachronous ILNM (occurring after surgery), or did not eventually undergo TME with curative intent.

### Neoadjuvant and adjuvant therapy

Patients with LARC who were being treated at our hospital with curative intent usually underwent nCRT followed by TME, either with or without adjuvant chemotherapy (ACT). The chemotherapy portion of nCRT consisted of induction (before radiotherapy), concurrent (during radiotherapy), and/or consolidation (after radiotherapy) regimens. The regimens chosen for each patient were determined at the discretion of the multi-disciplinary cancer team at our hospital. Chemotherapy typically involved a fluoropyrimidine-based regimen, consisting of one or more of the following: folinic acid with fluorouracil (de Gramont) and oxaliplatin (FOLFOX); capecitabine with oxaliplatin (CAPOX), or capecitabine (Xeloda) alone. Concurrent chemotherapy regimens always included oral capecitabine.

All patients received intensity-modulated radiotherapy (IMRT), and they were treated 5 days a week with 1 fraction daily. The radiotherapy was provided as follows: a total dose of 50 Gy to the planning target volume (PTV) of the gross tumor volume of the primary rectal tumor (GTVp), given in 25 fractions at 2.0 Gy per fraction; 45 Gy to the PTV of the clinical target volume (CTV), which always included the bilateral groins, given in 25 fractions at 1.8 Gy per fraction; and a simultaneous integrated boost (SIB) of 58 Gy in 25 fractions at 2.32 Gy per fraction (EQD_2_ = 59.55 Gy) to the PTV of the gross tumor volume of any clinically positive ILNs and lateral pelvic lymph nodes (GTVn). The CTV was defined as the GTV plus areas considered at significant risk of harboring microscopic disease, including the mesorectum (perirectal fascia), perirectal nodes, presacral region, and internal iliac lymph node region. External iliac nodes were included if the primary tumor invaded adjacent organs (cT4) or if the obturator nodes or external iliac nodes were involved [8].

A majority of the patients in this study also received fluoropyrimidine-based ACT, which was given after surgery and was also chosen at the discretion of the multi-disciplinary cancer team at our hospital. This team also determined how many cycles of neoadjuvant chemotherapy (NCT) and ACT were given to each patient. All patients in this study received a total of at least 8 chemotherapy cycles.

### Inguinal lymph node dissection (ILND)

All patients had repeat imaging after nCRT and before surgery. An ipsilateral superficial ILND was performed at the time of TME if imaging results were suspicious for residual disease in the ILNs. Suspicious ILNs were defined as those with a diameter 8 mm or larger on the short axis on CT or MRI, or with irregular borders and mixed signal intensity on MRI.

### Population characteristics

Demographic and baseline clinical characteristics evaluated in this study included the following: gender, age, tumor differentiation, clinical TNM stage, dentate line invasion, distance of distal tumor from anal verge, and clinical status of mesorectal and/or lateral pelvic lymph nodes. Clinicopathological characteristics evaluated in this study included the following: pathologic stage after neoadjuvant therapy and TME surgery (ypTN stage); pathologic complete response (pCR), defined as the absence of viable adenocarcinoma cells in the TME surgical specimen (ypT0N0); and surgical specimen pathology results (vascular invasion, neural invasion, surgical margin, and circumferential resection margin).

### Follow-up

Follow-up duration was defined as the time from the first day of any treatment to either the date of last examination or the date of death. Patients were routinely assessed at 3-month intervals during the first 3 years and at 6-month intervals thereafter. Treatment toxicities were recorded.

### Outcomes

The primary outcomes measured in this study were survival metrics and included the following: overall survival (OS), the time from the initiation of treatment to death from any cause, or, when censored, at the latest date if still alive; disease-free survival (DFS), the time from surgery to the first relapse at any site, death, or, when censored, at the latest date if still alive; distant metastasis-free survival (DMFS), the time from diagnosis to the first distant relapse (recurrence outside the pelvis and groins), or death, or when censored, at the latest date if still alive; and locoregional relapse-free survival (LRFS), the time from diagnosis to the first locoregional relapse (recurrence within the pelvis or groins), or death or, when censored, at the latest date if still alive.

### Statistical methods

The categorical variables are presented as frequencies; percentages were not used given the small sample size. ILNM measurements and radiation target volumes are presented as medians and ranges. All survival outcome measures were censored on May 1, 2021. Kaplan–Meier survival curves were used to calculate OS, DFS, LRFS, and DMFS rates. All statistical analyses were performed using SPSS, version 24.0 (SPSS, Inc., Chicago, IL, USA).

## Results

### Demographic and clinical characteristics

The median age of the 16 patients was 54 (range, 37 to 70) years (Table 1). The primary tumor was well- or moderate-differentiated in 12 patients. Of the 16 patients, 10 had clinical T3 stage and 6 had clinical T4 stage rectal cancer. A total of 6 patients had clinical N1 stage and 10 patients had clinical N2 stage. The tumor was within 10 cm of the anal verge in all patients and within 5 cm of the anal verge in 10 patients. All patients had clinical evidence of mesorectal lymph node metastasis, and 7 patients had evidence of lateral pelvic lymph node metastasis. All patients received at least 5 cycles of NCT, 13 received between 5 and 8 cycles of NCT, and 3 received between 9 and 12 cycles of NCT. After surgery, 13 of the 16 patients received ACT, and 7 of those received 5 or more cycles.

**Table 1:** Demographic and baseline clinical characteristics of 16 patients with LARC and synchronous SILNM

Characteristics	Patients n
Gender	
Male	8
Female	8
Age, years	
< 54	7
≥ 54	9
Tumor differentiation	
Well	2
Moderate	10
Poor	4
Clinical T stage	
T3	10
T4	6
Clinical N stage	
N1	6
N2	10
Dentate line invasion	
Positive	8
Negative	8
Distance distal tumor from anal verge, cm	
0–5	10
> 5 and ≤ 10	6
Mesorectal lymph node status	
Positive	16
Negative	0
Lateral pelvic lymph node status	
Positive	7
Negative	9
Neoadjuvant chemotherapy, cycles	
> 4 and ≤ 8	13
> 8 and ≤ 12	3
Adjuvant chemotherapy, cycles	
0	3
1–4	6
≥ 5	7

Demographic and baseline clinical characteristics of 16 patients who were diagnosed with LARC between January 2017 and December 2019, who had synchronous SILNM and no distant metastases, who underwent nCRT with ILN radiation boost prior to TME, and some of who underwent ACT. Clinical characteristics (including staging) based on MRI, EUS, and/or CT

LARC, locally advanced rectal cancer; SILNM, solitary inguinal lymph node metastasis; nCRT, neoadjuvant chemoradiotherapy; TME, total mesorectal excision; ACT, adjuvant chemotherapy; MRI, magnetic resonance imaging; EUS, endorectal ultrasonography; CT, computed tomography

### ILNM and radiation target characteristics

Of 16 patients, 7 had bilateral ILNM (Table 2). For the group, the median longest ILN diameter was 1.4 (range, 1.0–4.0) cm, and the median shortest diameter was 1.0 (range, 0.8–2.3) cm. The median GTV of ILNs was 29.4 (range, 13.6–183.7) cc, and the median CTV was 1750.9 (range, 1332.2–2200.5) cc.

**Table 2:** ILN metastasis and radiation target volume characteristics in patients with LARC and synchronous SILNM

Characteristic	Patients (N = 16)
Site of ILN metastasis, *n*	
Unilateral	9
Bilateral	7
Longest diameter of ILN, median (range), cm	1.4 (1.0–4.0)
Shortest diameter of ILN, median (range), cm	1.0 (0.8–2.3)
GTV of primary rectal cancer, median (range), cc	129.3 (64.3–279.7)
GTV of ILNs, median (range), cc	29.4 (13.6–183.7)
CTV, median (range), cc	1750.9 (1332.2–2200.5)
Volume of small bowel, median, range, cc	837.5 (382.6–1443.4)

Characteristics of the ILN metastases (based on pre-treatment MRI/CT) and radiation target volumes of 16 patients who were diagnosed with LARC between January 2017 and December 2019, had synchronous SILNM and no distant metastases, and underwent nCRT with ILN radiation boost prior to TME. ILN characteristics based on MRI, EUS, and/or CT

ILN, inguinal lymph node; LARC, locally advanced rectal cancer; SILNM, solitary inguinal lymph node metastasis; GTV, gross tumor volume; CTV, clinical target volume; MRI, magnetic resonance imaging; CT, computed tomography; nCRT, neoadjuvant chemoradiotherapy; TME, total mesorectal excision; EUS, endorectal ultrasonography

### Clinicopathological characteristics

All patients underwent TME with curative intent. A total of 5 patients underwent ipsilateral superficial ILND at the time of TME because of suspicious ILN findings on imaging (3 patients with ILN 8 mm or larger on CT or MRI, and 2 patients with ILN that had irregular borders and mixed signal intensity on MRI). Of those 5 patients, 2 were ultimately found to have residual tumor present in those lymph nodes (Table 3). The median number of lymph nodes obtained in each dissection was 14 (range, 8–21). Of the 16 patients, 3 were stage ypT0 and 8 were stage ypN0. In total, 3 patients were stage ypT0N0, achieving a pathological complete response (pCR). All patients had negative surgical margin except one had circumferential resection margins lesser than 1 mm.

**Table 3:** Postoperative clinicopathological characteristics in 16 patients with LARC and synchronous SILNM

Characteristics	Patients n
ILN dissection (after radiotherapy)	5
ILN positive for residual tumor	2
Pathological T stage	
ypT1	1
ypT2	5
ypT3	4
ypT4	3
Pathological N stage	
ypN0	8
ypN1	5
ypN2	3
Pathological TNM stage	
ypT0N0	3
I	2
II	4
III	7
Pathologic complete response (pCR)	
Yes	3
No	13
Microvascular invasion	
Negative	13
Positive	3
Neural invasion	
Negative	15
Positive	1
Surgical margin	
Negative	16
Positive	0
Circumferential resection margin, mm	
≤ 1	1
> 1	15

Clinicopathological characteristics of 16 patients who were diagnosed with LARC between January 2017 and December 2019, who had synchronous SILNM and no distant metastases, who underwent nCRT with ILN radiation boost prior to TME, and some of who had ILN dissection during TME. The yp stage represents the pathologic stage after neoadjuvant therapy and surgical resection

LARC, locally advanced rectal cancer; SILNM, solitary inguinal lymph node metastasis; ILN, inguinal lymph node; nCRT, neoadjuvant chemoradiotherapy; TME, total mesorectal excision

### Survival results and failure patterns

**Fig. 1:**
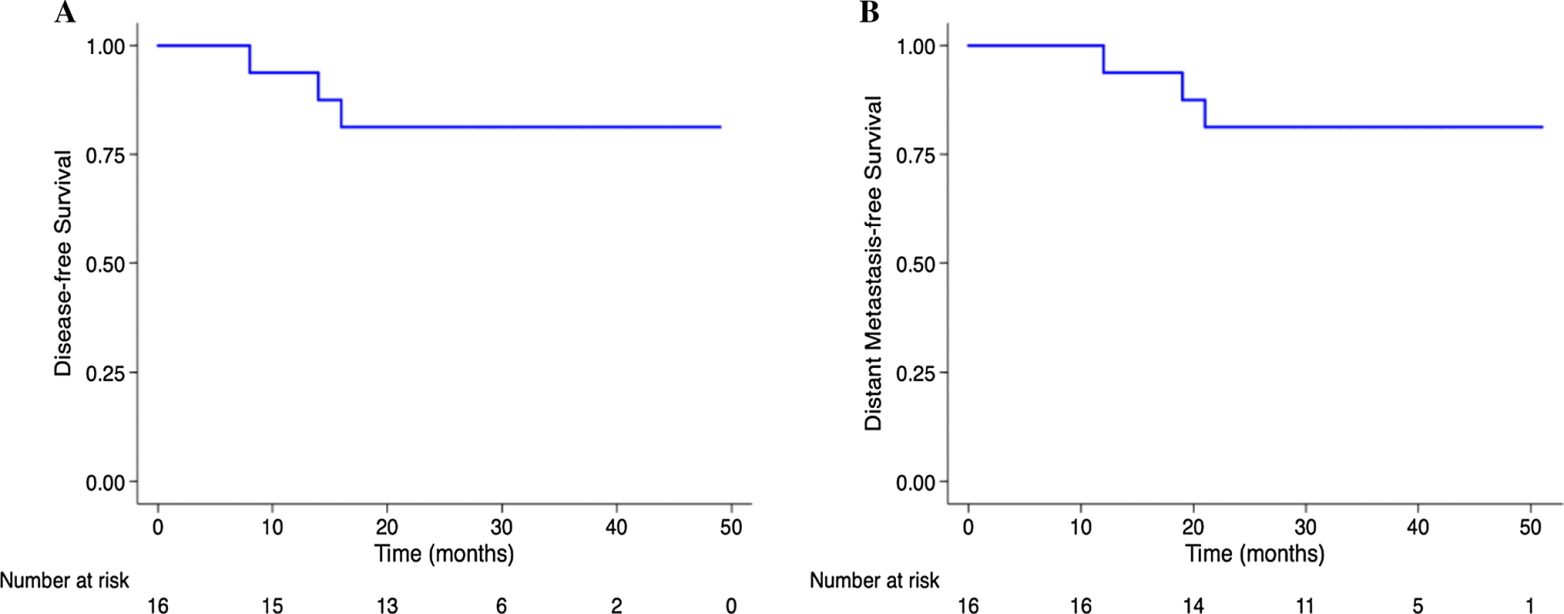
Kaplan–Meier analysis curves of 16 patients who were diagnosed with LARC between January 2017 and December 2019, who had synchronous SILNM and no distant metastases, who underwent nCRT with ILN radiation boost prior to TME, some of who underwent ILN dissection, and some of who received ACT: **A** disease-free survival (DFS), and **B** distant metastasis-free survival (DMFS). The 3-year DFS and DMFS rates were both 81.3%. LARC, locally advanced rectal cancer; SILNM, solitary inguinal lymph node metastasis; nCRT, neoadjuvant chemoradiotherapy; TME, total mesorectal excision; ACT, adjuvant chemotherapy

The median follow-up for all patients was 36.5 (range, 29–51) months. All patients were alive at the last follow-up, so the 3-year OS rate was 100%. No patients exhibited local recurrence, including in the pelvis or groins, so the 3-year LRFS rate was also 100%. The 3-year DFS and DMFS rates were both 81.3% (Fig. 1). A total of 3 patients had disease progression based on evidence of distant metastases: lung metastases in one patient, bone metastases in one patient, and left supraclavicular and para-aortic lymph node metastases with bone metastases in one patient (Table 4).

**Table 4:** Individual demographic, baseline clinical, and clinicopathological characteristics of 16 patients with LARC and synchronous SILNM

Patient Number	Age, y*ears*	Sex	Rectal Cancer Clinical Stage	Distance from anal verge, *cm*	Dentate Line Invasion	ILN metastasis Location	Longest ILN diameter (baseline)	Shortest ILN diameter (baseline)	Longest ILN Diameter	Shortest ILN Diameter	ILN Dissection	Residual ILNM Confirmed	Follow up
									After nCRT	After nCRT	ILN Dissection		
1	65	M	T4N1	6.7	No	Right	2.3	1.1	NR	NR	NO	NA	Alive, NED
2	63	F	T3N1	3.5	No	Right	1.2	1.0	NR	NR	NO	NA	Alive, NED
3	54	F	T3N2	2.5	Yes	Left	1.2	0.8	1.0	0.8	YES	NO	Alive, NED
4	43	F	T4N1	1.9	Yes	Bilateral	1.4	0.8	0.7	0.5	NO	NA	Alive, NED
5	61	M	T3N2	2.2	Yes	Bilateral	2.0	0.9	NR	NR	NO	NA	Alive, NED
6	54	M	T3N2	0.5	Yes	Bilateral	4.0	1.7	1.2	0.8	YES	YES	Alive, NED
7	55	F	T4N2	5.0	No	Bilateral	3.5	2.3	1.9	1.2	YES	YES	Alive, NED
8	44	F	T3N1	3.0	Yes	Bilateral	1.2	1.0	NR	NR	NO	NA	Alive, NED
9	63	M	T3N2	5.7	No	Bilateral	1.4	1.1	NR	NR	NO	NA	Alive, PD
10	66	F	T4N1	0.0	Yes	Right	1.2	1.0	NR	NR	NO	NA	Alive, PD
11	38	F	T3N2	2.9	Yes	Left	1.4	1.2	0.8	0.7	NO	NA	Alive, NED
12	46	M	T3N1	3.2	No	Left	1.1	0.8	NR	NR	NO	NA	Alive, NED
13	37	M	T4N2	5.1	No	Left	1.0	0.8	NR	NR	NO	NA	Alive, PD
14	48	M	T4N2	5.1	No	Bilateral	1.2	1.0	NR	NR	NO	NA	Alive, NED
15	36	F	T3N2	5.6	No	Right	1.4	1.0	0.7	0.5	YES^b^	NO	Alive, NED
16	70	M	T2N2	2.5	Yes	Right	2.8	1.4	1.0	0.7	YES^b^	NO	Alive, NED

Individual characteristics of 16 patients who were diagnosed with LARC between January 2017 and December 2019, who had synchronous SILNM and no distant metastases, who underwent nCRT with ILN radiation boost prior to TME, some of who had ILN dissection during TME, and some of who received ACT

LARC, locally advanced rectal cancer; SILNM, solitary inguinal lymph node metastasis; ILN, inguinal lymph node; nCRT, neoadjuvant chemoradiotherapy; TME, total mesorectal excision; ACT, adjuvant chemotherapy; NR, no residual; NA, not applicable; NED, no evidence of disease; PD, progression of disease; MRI, magnetic resonance imaging; CT, computed tomography

^a^Ipsilateral superficial ILN dissection performed for suspicious ILNs, which were based on MRI or CT done after NCRT, and which were defined as those with a diameter 8 mm or larger on the short axis on MRI or CT, or with irregular borders and mixed signal intensity on MRI

^b^Patient had ILN with irregular borders and mixed signal intensity on MRI

### Radiation-related toxicity

Toxicities that were observed correlated clinically with the radiation boost to the ILNs. The majority of toxicities were Grades 1 and 2, including inguinal dermatitis and inguinal edema. Grade 3 radiation enteritis was observed in 2 patients, both of whom underwent ILND after radiation boost to the ILNs. Grade 3 anastomotic inflammation occurred in 3 patients, 2 of whom underwent ILND after radiation boost to the ILNs. No lymphedema or anastomotic fistula, and no Grade 4 or 5 toxicities, were observed.

## Discussion

The results of this case series of 16 patients with LARC who had synchronous SILNM suggests that a radiation boost to ILNs with curative intent, given as part of nCRT, and followed by TME, is associated with excellent intermediate-term local control and low toxicity. None of the patients in this cohort died or had an ILN or pelvic recurrence during a median follow-up of 3 years. Only 3 of the patients eventually had disease progression with distant metastases, primarily to the lung and bone. Toxicities possibly attributable to the radiation boost were mostly grade 1 or 2, and none of the toxicities in our cohort were life-threatening or disabling.

SILNMs from rectal cancer, defined as ILNM without evidence of other systemic metastases, are rare. They are generally considered to represent systemic disease, owing to their association with poor prognosis and distant metastases [1, 9–11]. However, reports suggest that patients with SILNMs should be treated as a distinct subset of those with systemic disease, in part because ILND has resulted in prolonged survival and even cure in some of these patients [2, 6, 10, 12]. Graham and Hohn reported on a series of 8 patients who underwent abdominoperineal resection (APR) and then develop metachronous SILNM [4]. Each patient then underwent ILND as salvage treatment. Of the 8 patients, 6 survived more than 2 years. Hagemans et al. retrospectively reviewed 27 patients with ILNM with rectal cancer, of which 12 patients had SILNM and underwent ILND with curative intent. The median duration of OS for these 12 patients was 74 months, and their 1- and 5-year estimated OS rates were 83% and 52%, respectively [2].

Patients in our study received an ILN radiation boost (during nCRT) for their SILNM, as an alternative to surgical ILN dissection. Not much has been published about either approach for the treatment of SILNM. Yeo et al. reported on 3 patients with LARC who were treated with curative intent and then developed isolated ILN recurrences [11]. The authors subsequently performed salvage therapy with curative intent, using concurrent CRT in 2 patients and surgical excision followed by concurrent CRT in 1 patient. One patient was alive with no evidence of disease 10 years after ILN recurrence and the other two patients (one of whom had the surgical excision) had no ILN recurrence for over 5 years but did develop pelvic and distant recurrences or a secondary malignancy. In our study with shorter follow-up, none of the patients treated with an ILN radiation boost developed an ILN recurrence, resulting in a 3-year LRFS rate of 100%. At the same time, 2 patients failed inguinal lymph node control after nCRT had the largest tumor sizes, in which the longest ILN diameter was 3.5 cm and 4 cm, respectively (Table 4). This may suggest that our current prescribed dose is insufficient for large ILN. Higher radiation dose is associated with a higher probability of pathological tumor regression [13]. A systematic review on the efficacy of dose escalation to ≥ 60 Gy in LARC, a higher pooled pCR rate was achieved compared with standard chemoradiation (20.4% vs. 10.3%) [14]. Nevertheless, the RECTAL-BOOST, a phase 2 randomized controlled trail, explored the efficacy of external radiation boost (5 × 3 Gy) followed by standard pelvic chemoradiation, which demonstrate dose escalation did not increased pCR rate or sustained clinical complete response in LARC, compared with standard nCRT [15]. The efficacy of optimal radiation dose for positive ILN remains unclear. In addition, only 3 patients developed distant metastases, so that the 3-year DFS rate for the patients in our cohort was 81.3%. Longer follow-up of our patients and additional studies comparing ILND to ILN radiation boost for SILNM are needed to determine if these 2 approaches are comparable. More recently, new treatment strategies have been used for LARC in an attempt to address the predominant cause of failure, which is distant relapse. These strategies have focused on giving more cycles of chemotherapy prior to TME, either as induction chemotherapy prior to nCRT or as consolidation chemotherapy after radiation [12, 16]. In a meta-analysis involving 28 studies, high DFS and OS rates were observed for patients receiving induction chemotherapy, nCRT, and/or consolidation chemotherapy prior to TME for LARC [17]. In our study, all patients received at least 5 cycles of NCT (either as induction or consolidation) prior to surgery, which certainly may have played a role in the relatively high level of disease control. Thus, the best treatment algorithm for patients presenting with LARC and synchronous SILNM still needs to be clarified.

Extending the radiation field to encompass the groin regions can be associated with a variety of acute and delayed morbidities, including dermatitis or fibrosis of the inguinal skin, wound and bowel complications, lower extremity edema, and femoral neck fractures [18]. At the same time, ILND has its own substantial adverse sequelae, including lymphedema, deep venous thrombosis (DVT), wound infection, skin necrosis, lymphocele, and seroma [19]. Lee and colleagues performed elective ILN radiation in 164 patients with pelvic tumors suspected to be at significant (> 10%) risk of harboring occult disease in the ILNs [20]. Their study demonstrated that ILN radiation resulted in fewer treatment-related complications and shorter hospital stays than have historically been reported in studies involving ILND. Shiratori et al. reported on 145 patients with lower rectal cancer, including 7 with SILNM, 17 with metachronous ILNM (MILNM), and 121 without ILNM [21]. They noted that of 8 patients treated with ILND alone, 1 (13%) patient developed persistent ipsilateral leg lymphedema; of 12 patients receiving only ILN radiation, none developed lymphedema; and of 7 patients undergoing ILN radiation with ILND, 2 (29%) patients developed persistent ipsilateral leg lymphedema.

The combination of ILND and groin irradiation may increase the morbidity for patients with SILNM from rectal cancer beyond that caused by either treatment alone [2, 21]. Hageman and colleagues reported on 17 patients with rectal cancer who underwent ILND with curative intent [2]. One of these patients received neoadjuvant ILN radiation, and the rest had the inguinal areas at least partly included in the nCRT radiation field. A total of 6 patients subsequently experienced substantial complications, including inguinal seroma requiring percutaneous drainage, superficial wound infections requiring antibiotics, and persistent lower extremity lymphedema requiring elastic compression garments. In the population reported by Shiratori et al. and described above, the rate of lymphedema was the highest (29%) when patients underwent both radiation and ILND [21]. In our study, although 5 patients received ILND after radiation, no patients were identified with lymphedema, anastomotic fistulae, or grade 4–5 radiation toxicity. This may in part be because all patients in our cohort received IMRT, which has demonstrated dosimetric advantages as well as decreased toxicity from groin irradiation [22, 23]. Although these results suggest lower levels of toxicity when compared to previous studies, it remains to be determined with a larger prospective trial whether ILN boost radiation can reliably achieve adequate disease control while also offering acceptable complication rates.

There are several limitations to the study that should be acknowledged. First, this is a single-arm retrospective non-controlled study from a single institution, with treatment regimens that varied and were determined at the discretion of our multi-disciplinary cancer team, all of which may have resulted in selection bias and confounding. Second, the cohort in this study was small, primarily because of the rarity of SILNM from rectal cancer. Consequently, a multi-center, prospective, controlled study would be needed to accrue a larger population, address some of the bias, and validate our findings. This would help to establish the optimal treatment strategy for SILNM from LARC. Third, the follow-up period was not long enough to adequately evaluate disease progression rates and radiation-related chronic toxicities. However, the results of the study remain encouraging given that the shortest follow-up was 29 months, the median follow-up was more than 3 years, and none of the patients developed inguinal recurrences.

## Conclusion

In patients with LARC and synchronous SILNM, a radiation boost to the ILNs during nCRT achieved excellent local control with acceptable toxicity. Though the optimal treatment strategy remains unclear, nCRT with an ILN radiation boost prior to TME may be a reasonable therapeutic approach to consider for this subset of patients.

## Data Availability

The datasets used for this current study are available from the corresponding author upon reasonable request.
